# Cytomegalovirus-induced cutaneous ulcers: A case series

**DOI:** 10.1016/j.jdcr.2025.04.016

**Published:** 2025-04-26

**Authors:** Shruti Agrawal, Seneca D. Hutson, Emma F. Johnson, Afsaneh Alavi

**Affiliations:** a Department of Dermatology, Mayo Clinic, Rochester, Minnesota; b Department of Laboratory Medicine and Pathology, Mayo Clinic, Rochester, Minnesota

**Keywords:** CMV, immunocompromised, mucocutaneous, ulcer

## Introduction

The human herpes virus (HHV) family encompasses a broad range of pathogens and are well-documented causes of skin lesions, including herpes simplex virus (HSV), varicella zoster virus (VZV), Epstein-Barr virus (HHV-4), HHV-6 and HHV-7 (implicated in drug reaction with eosinophilia and systemic symptoms syndrome), HHV-8 (known to cause Kaposi sarcoma), and cytomegalovirus (CMV, HHV-5).

CMV is a double-stranded deoxyribonucleic acid (DNA) virus that affects a large portion of the global population, estimated to have infected 60% to 70% of those in industrialized countries and nearly 100% in emerging countries.^1^ CMV infections are generally asymptomatic or mild in immunocompetent individuals, with clinical manifestations possibly including rash, fever, and leukocytosis.^1^ CMV remains dormant over the life of the host in myeloid cells, reactivating in the setting of immunosuppression.^1^ Infection in immunocompromised hosts can cause life-threatening manifestations in a variety of organ systems, potentially causing retinitis, colitis, hepatitis, pneumonia, myocarditis, encephalitis, neuritis, and meningitis.^1^ The growing population of immunocompromised individuals raises concerns about susceptibility to infections with atypical presentations and prolonged illnesses.

Cutaneous manifestations of CMV have been previously reported, with ulceration representing the most common presentation.^2^ Other cutaneous findings reported in the literature include necrotizing vasculitis,^3^ thrombosis leading to limb ischemia,^4^ Degos-like skin lesions,^5^ and drug reaction with eosinophilia and systemic symptoms/drug-induced hypersensitivity syndrome,^6^ among others.

We present a case series of 4 immunocompromised individuals presenting with CMV cutaneous ulcerations with evidence of CMV viremia and positive CMV polymerase chain reaction (PCR) swabs. Available histopathologic data are also included.

## Materials and methods

After approval by our organization’s Institutional Review Board, a search was performed of all skin biopsy reports performed from 2000 to 2023 at our institution containing “cytomegalovirus.” 3 specimens from 2 patients were identified. 2 cases of CMV cutaneous ulceration were identified prospectively.

## Case series

### Case #1

**Fig 1:**
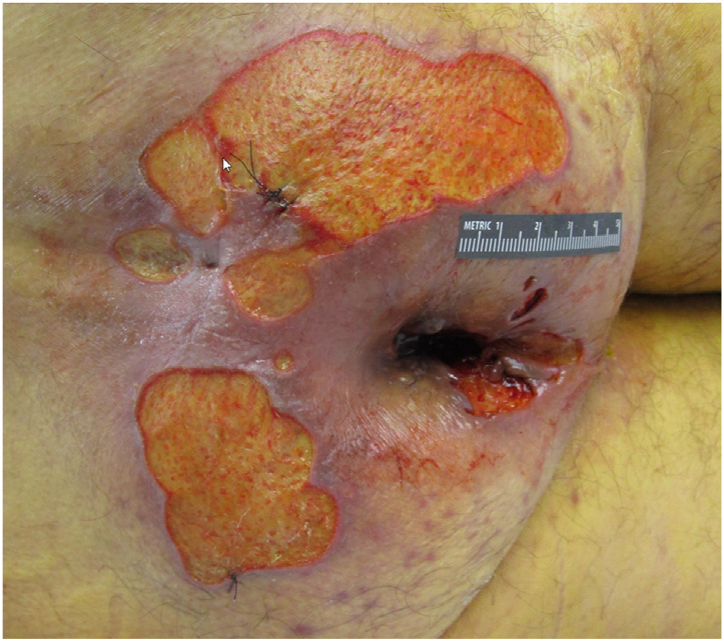
Cytomegalovirus-induced cutaneous ulcers. Patient #1 presenting with multiple well-demarcated polycyclic ulcerations on the perianal region.

A 71-year-old male with a history of Hodgkin’s lymphoma and primary biliary cirrhosis was admitted for constrictive pericarditis secondary to malignant mesothelioma. Dermatology was consulted for a 6-month history of nonhealing ulcers, with examination demonstrating polycyclic, well-demarcated sacral and buttock ulcerations (Fig 1).

**Fig 2:**
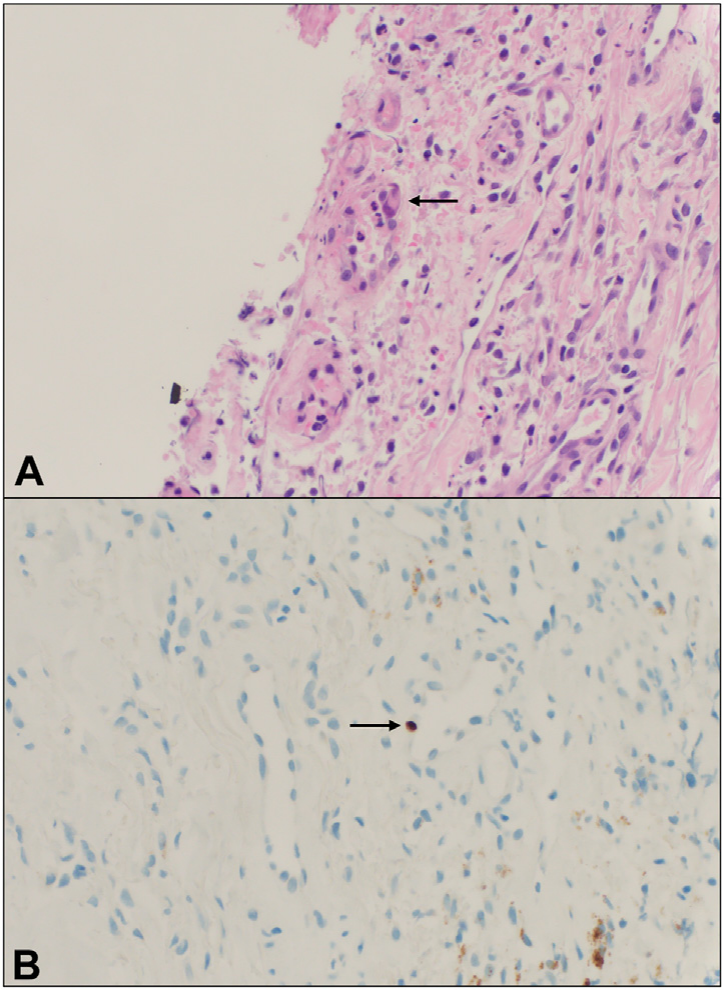
Cytomegalovirus-induced cutaneous ulcers. Histopathologic evaluation shows rare endothelial cells with nuclear enlargement and areas suspicious for viral cytopathic change (**A,** Hematoxylin & eosin [H&E] stain; 200×) with positive CMV immunohistochemical (IHC) staining in affected endothelial cells (**B,** CMV IHC; 200×). Arrow marks show rare endothelial cells with nuclear enlargement (**A**), with corresponding positivity on CMV IHC staining (**B**). *CMV*, Cytomegalovirus.

Clinical suspicion was highest for herpetic infection, with superimposed changes secondary to poor nutritional status, stool incontinence, and prolonged debility. HSV and VZV PCR swabs were negative. CMV PCR swab was positive. 2 punch biopsies were subsequently performed, demonstrating ulceration with acute inflammation, angioplasia, and rare enlarged endothelial cells with viral changes suspicious for CMV infection (Fig 2, *A*). CMV immunohistochemical (IHC) staining was positive in rare endothelial cells of blood vessels underlying the ulceration (Fig 2, *B*).

CMV DNA PCR was performed on serum, demonstrating a viral load of 21,700 IU/mL. The patient was treated with intravenous (IV) ganciclovir, with improvement of viral load to 342 IU/mL over approximately 17 days. The patient’s clinical course was complicated by septic shock and multiple organ dysfunction, and the patient subsequently expired.

### Case #2

**Fig 3:**
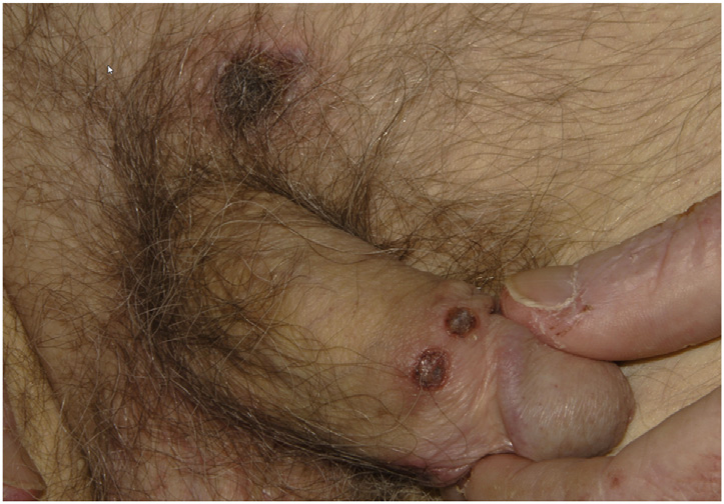
Cytomegalovirus-induced cutaneous ulcers. Patient #2 presenting with multiple well-demarcated circular ulcerations with hemorrhagic crust on the suprapubic area and penis.

A 55-year-old male with a history of a liver transplant for primary sclerosing cholangitis and cholangiocarcinoma, on mycophenolate mofetil, tacrolimus, and prednisone, presented with fever, dysphagia, a full body rash composed of pink atrophic macules, and localized ulcerations affecting the suprapubic area and penis (Fig 3), clinically suspected to represent herpes simplex infection.

**Fig 4:**
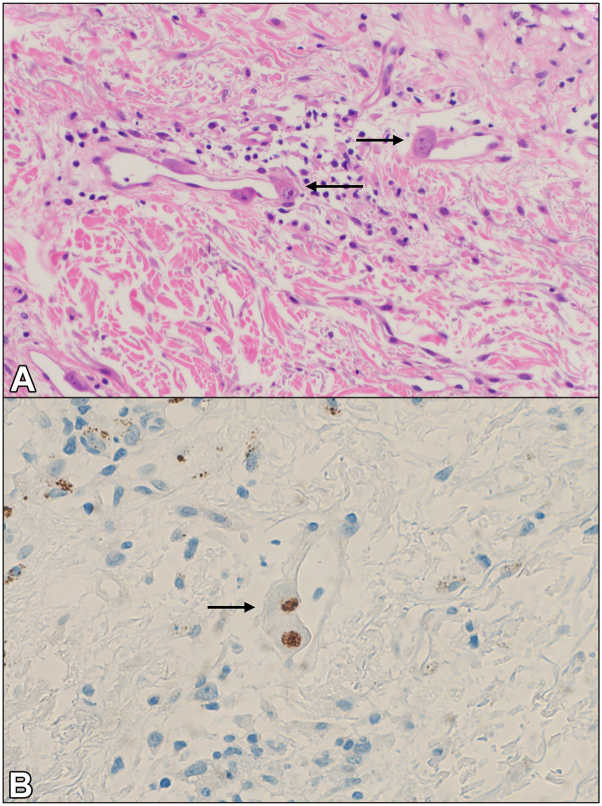
Cytomegalovirus-induced cutaneous ulcers. Histopathologic examination of biopsy from a rash from the shoulder of Patient #2 shows endothelial cells with cytologic atypia including nuclear enlargement and prominent nucleoli (**A,** H&E, 200×) with positive CMV IHC staining (**B,** CMV IHC, 200×). Arrow marks show rare endothelial cells with nuclear enlargement (**A**), with corresponding positivity on CMV IHC staining (**B**). *CMV*, Cytomegalovirus; *H&E*, hematoxylin & eosin; *IHC*, immunohistochemical.

Esophagogastroduodenoscopy was performed, with pathology of the esophagus, stomach, and duodenum demonstrating numerous CMV inclusions. Given the presence of widespread rash, a punch biopsy of the right shoulder was performed to rule out a drug rash. Pathology demonstrated vacuolar interface changes of the epidermis, in addition to endothelial viral inclusions with positive CMV IHC staining (Fig 4).

Direct immunofluorescence testing was negative. Swab of the genital ulcerations was positive for HSV and CMV, both by PCR. The patient was treated with IV ganciclovir, resulting in improvement of the rash. Serum quantitative CMV DNA PCR originally demonstrated a viral load of 2,373,500 IU/mL, which improved to 3500 IU/mL with treatment over approximately a month. Follow-up information regarding status of the ulcerations was not available. The patient subsequently expired secondary to pneumonia.

### Case #3

**Fig 5:**
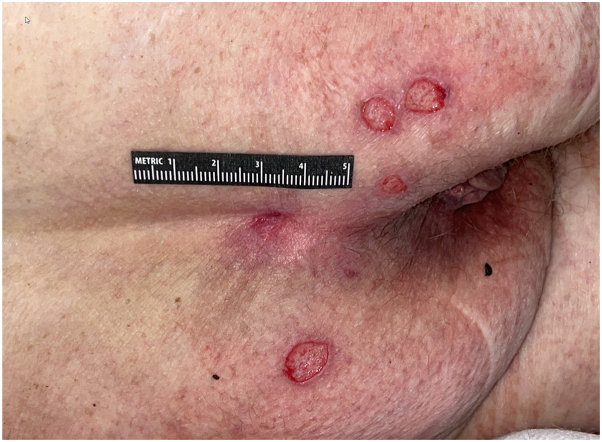
Cytomegalovirus-induced cutaneous ulcers. Patient #3 presenting with multiple superficial circular ulcerations on the buttocks and gluteal cleft.

A 69-year-old female with a history of non-small cell lung cancer, treated with lobectomy, carboplatin, and pemetrexed, was admitted originally for myocarditis and subsequently for perforated colon. Dermatology was consulted on both admissions for multiple superficial ulcerations affecting the buttocks and gluteal cleft (Fig 5).

**Fig 6:**
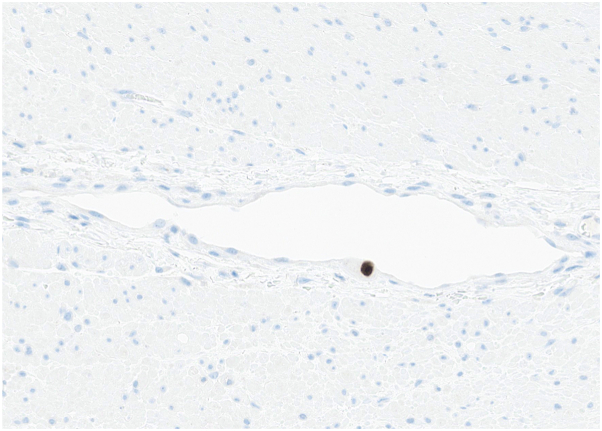
Cytomegalovirus-induced cutaneous ulcers. Pathology specimen from the colon demonstrating positive CMV IHC staining within endothelial cells (CMV IHC, 200×). *CMV*, Cytomegalovirus; *IHC*, immunohistochemical.

PCR swabs for HSV and VZV were negative. Given high clinical suspicion for herpetic infection, prophylactic oral valacyclovir was started without improvement in ulcerations. CMV PCR swab of the buttocks ulcerations was subsequently performed and was positive. Serum CMV DNA quantification demonstrated a viral load of 12,000 IU/mL which improved to undetectable levels over a month with treatment with IV valganciclovir. Skin biopsy was not performed. However, biopsies of the colon were performed given the concurrent gastrointestinal symptoms, with pathology demonstrating positive CMV staining by immunohistochemistry of stromal and endothelial cells (Fig 6). The cutaneous and gastrointestinal symptoms improved with treatment.

### Case #4

**Fig 7:**
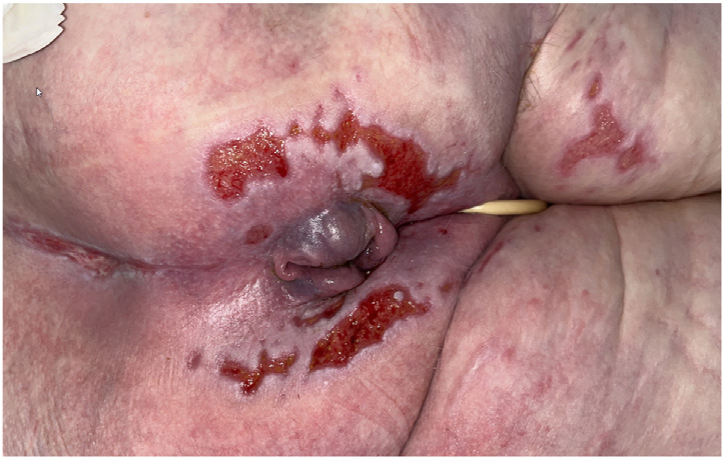
Cytomegalovirus-induced cutaneous ulcers. Patient #4 presenting with multiple angulated ulcerations on the buttocks.

A 75-year-old female with a history of colon adenocarcinoma and breast cancer was admitted for coronavirus disease 2019 infection and pneumocystis pneumonia. Dermatology was consulted for painful angulated ulcerations of the buttocks and gluteal cleft (Fig 7), with clinical suspicion for viral infection and possible irritant contact dermatitis due to incontinence.

CMV and HSV swabs were positive, while swabs for VZV and enterovirus were negative. The positive CMV swab was considered diagnostic, and no skin biopsy was obtained. She was started on IV ganciclovir for a treatment course of 14 days with improvement in ulcerations. Serum CMV DNA quantification improved from 2700 to 66 IU/mL. After goals of care discussion, the patient decided to pursue hospice treatment and subsequently expired.

Patient demographics, clinical findings, and laboratory studies are summarized in Table I.

**Table I:** Demographic and characteristics of patients presenting with CMV-induced ulcerations

Case	Age (y)	Sex	Pertinent medical history	Ulcer location	Morphology	HSV swab result	CMV PCR swab result	CMV viral load (IU/mL)	Treatment
1	71	M	Hodgkin lymphoma, primary biliary cirrhosis, malignant mesothelioma	Sacrum, buttocks	Polycyclic, well-demarcated, superficial ulcers	Negative	Positive	21,700-342	IV ganciclovir
2	55	M	Liver transplant for primary sclerosing cholangitis and cholangiocarcinoma	Suprapubic, penis	Superficial, well-demarcated round ulcers	Positive	Positive	2,373,500-3500	IV ganciclovir
3	69	F	Non-small cell lung cancer	Buttocks	Superficial, well-demarcated round ulcers	Negative	Positive	12,000-undetectable	IV valganciclovir
4	75	F	Colon adenocarcinoma, breast cancer	Buttocks	Superficial, well-demarcated angulated ulcerations	Positive	Positive	2700-66	IV ganciclovir

*CMV*, Cytomegalovirus; *F*, female; *HSV*, herpes simplex virus; *IV*, intravenous; *M*, male; *PCR*, polymerase chain reaction.

## Discussion

CMV infections can cause life-threatening disease in immunocompromised patients, leading to systemic and cutaneous manifestations. CMV cutaneous ulcerations should be considered in the differential diagnosis of anogenital ulcerations in immunocompromised patients. As seen in this case series, all 4 patients were immunocompromised, and 3 of the 4 patients subsequently expired soon after diagnosis of CMV infection. The potential severity of this infection highlights the importance of making a timely and accurate diagnosis.

Skin biopsy is a valuable tool to help make the diagnosis of CMV infection. Classic histopathologic features of CMV infection include the presence of viral cytopathic change within the dermis, largely within mesenchymal cells, such as endothelial cells, macrophages, or fibroblasts.^7^ Rarely, involvement of the eccrine duct epithelium and lumens has been noted.^7^ Specifically, endothelial cells and macrophages demonstrate nuclear enlargement and nuclear inclusions with a surrounding clearing, sometimes likened to “owl eyes”.^8^^,^^9^ Nuclear positivity by CMV IHC is helpful in confirming the diagnosis.^10^ In 1 series, only approximately one-third of cases demonstrated nuclear atypia, with a larger proportion demonstrating cytoplasmic changes.^9^ Resnik et al proposed that cytologic changes differ based on the stage of CMV infection. At first, affected cells demonstrate nuclear enlargement, then cytoplasmic changes, including a smudged or “bubbly” quality, followed by the development of nuclear and cytoplasmic inclusion bodies, which is thought to be a characteristic of a fully developed cutaneous CMV infection. As the infection resolves, the size of the cell and nucleus decreases, followed by resolution of cytoplasmic inclusions and fragmentation of nuclear inclusions.^7^

While entirely classic “owl eye” inclusions were not seen on the available biopsy specimens from case #1 or case #2, endothelial atypia with enlarged nuclei was identified in both cases. CMV IHC was also beneficial in highlighting affected endothelial cells. Interestingly, in case #2, the biopsy was not performed from the genital ulcerations, but was instead taken from a rash on the shoulder, with pathology showing concurrent vacuolar interface dermatitis and viral cytopathic changes in endothelial cells. This case demonstrates that skin biopsy even from sites distant of cutaneous ulceration may be beneficial in making a diagnosis of CMV infection if the patient has evidence of systemic viremia.

Skin biopsy specimens from the other 2 patients were not performed, and therefore histopathologic examination was not possible. However, the diagnosis was confirmed with positive CMV swabs from the wound beds. This case series demonstrates that CMV PCR swabs are potentially beneficial as an alternative method to make a diagnosis, even if skin biopsy is unable to be obtained. The clinical significance of a positive swab result can be uncertain. For example, in a series of incidentally detected external and mucosal positive CMV swab results, only 5% were deemed to be clinically relevant, all in immunosuppressed patients.^11^ However, in the current series, a positive CMV swab was seen in all 4 patients, all of which were also found to have CMV viremia. Therefore, in the context of a CMV swab taken directly from a cutaneous ulceration, the authors believe the significance of a positive result, especially in an immunocompromised patient, may be highly relevant. Given the noninvasive nature and quick turnaround time of this test, CMV swabs may be beneficial in at least suggesting the diagnosis and prompting evaluation for systemic disease. Further studies may be beneficial to determine the sensitivity and specificity of this test in this clinical context.

CMV ulcerations are most frequently seen in the genital, perianal, or gluteal region and less frequently in the oral mucosa.^2^ It has been proposed that there may be a predilection for ulcerations in the anogenital area given CMV can affect the colon and possibly lead to viral shedding.^12^ All 4 patients in this series presented with ulcerations in the anogenital region. Additionally, in both case #2 and case #3, pathology specimens from the gastrointestinal track demonstrated CMV positivity by immunohistochemistry. Although a skin biopsy was not performed in case #3, swab for CMV PCR from the buttock ulcers was positive, further supporting that viral shedding may lead to anogenital skin findings.

Co-infection with CMV and HSV has also been previously reported.^13^^,^^14^ In this series, 4 of 4 patients were evaluated for HSV, with 2 showing evidence of co-infection. Thus, a positive HSV swab does not exclude a concurrent diagnosis of CMV, which is important given the potential for extracutaneous complications.

In conclusion, it is important for dermatologists and dermatopathologists to be aware of the cutaneous manifestations of CMV and maintain a high suspicion for this infectious etiology in immunocompromised patients presenting with ulceration. In addition to performing skin biopsy, evaluating for viremia, and performing PCR swabs for CMV may represent additional diagnostic tools.

## Conflicts of interest

None disclosed.
